# Forward variable selection for random forest models

**DOI:** 10.1080/02664763.2022.2095362

**Published:** 2022-07-18

**Authors:** Jasper Velthoen, Juan-Juan Cai, Geurt Jongbloed

**Affiliations:** a Department of Applied Mathematics, Delft University of Technology, Delft, The Netherlands; b Department of Econometrics and Data Science, Vrije Universiteit Amsterdam, De Boelelaan 1105, Amsterdam 1081 HV, The Netherlands

**Keywords:** Random forests, variable selection, CRPS, forward selection, correlated covariates

## Abstract

Random forest is a popular prediction approach for handling high dimensional covariates. However, it often becomes infeasible to interpret the obtained high dimensional and non-parametric model. Aiming for an interpretable predictive model, we develop a forward variable selection method using the continuous ranked probability score (CRPS) as the loss function. eOur stepwise procedure selects at each step a variable that minimizes the CRPS risk and a stopping criterion for selection is designed based on an estimation of the CRPS risk difference of two consecutive steps. We provide mathematical motivation for our method by proving that in a population sense, the method attains the optimal set. In a simulation study, we compare the performance of our method with an existing variable selection method, for different sample sizes and correlation strength of covariates. Our method is observed to have a much lower false positive rate. We also demonstrate an application of our method to statistical post-processing of daily maximum temperature forecasts in the Netherlands. Our method selects about 10% covariates while retaining the same predictive power.

## 1. Introduction

In the past decades, random forests [2] have gained traction in many areas of application. Specifically in the last years, random forests have been successfully used for statistical post-processing of weather forecasts, e.g. [19,21] and [18]. A random forest combines several trees, each obtained by recursively making axis-aligned splits in the covariate space until a stopping criterion is reached. The initial algorithm for random forests in [2] provides a good approach for conditional mean regression and classification. Later on, the approach was extended to estimate quantiles by Meinshausen [14] and further improvements were made in [1], which introduced a quantile-based splitting criterion. Due to the results in [1,14], random forests are also used for estimating the conditional quantile function.

These quantile forests have been used in statistical post-processing to obtain probabilistic forecasts, e.g. [18,19,21]. Post-processing is used as a second step in weather forecasting following a first step of physical modeling, see [11]. This first step entails a numerical weather prediction (NWP) model that uses non-linear partial differential equations of atmospheric flow on a spatial and temporal grid. Together with parametrizations of unresolved physical processes within the grid cells and an estimated initial condition, which is obtained from observational data and a so called first guess (i.e. a forecast for that time based on a previous NWP model run), the NWP model approximates the solution to the partial differential equations. An ensemble prediction system (EPS) adds uncertainty quantification to the NWP model by computing an ensemble of forecasts for perturbed initial conditions and/or the parametrization schemes [11].

Generally there is still a need for bias correction and calibration of numerical weather forecasts, which motivates the second step: statistical post-processing. Historical forecasts together with the corresponding observations are used in post-processing to estimate their statistical relationship. This relationship can then be used in order to calibrate future forecasts.

When post-processing forecasts of a weather phenomenon, a better performance is often attained by adding more information from the NWP models as predictors. For example, [21] showed that the post-processed precipitation forecasts perform substantially better when indices of atmospheric instability from the NWP models are used in modeling the statistical relation. The improvement is due to the fact that the indices of atmospheric instability help to distinguish between different types of precipitation. A full day of drizzle might accumulate to the same amount as a quick shower. However, the distributions of precipitation under these two different weather conditions are very different. Incorporating NWP forecasts of other weather phenomena enables the model to capture such differences.

A natural question is now: ‘Which additional forecasts contain useful information on the phenomenon that one is post-processing?’ The set of potential forecasts to include in the statistical model is generally very large and furthermore they exhibit large correlations. In practice, including too many variables often leads to a decrease in statistical efficiency, and more importantly the model becomes hard to interpret. For a practitioner, it is important to understand which variables play key roles in the statistical model and how they calibrate the EPS forecast. This motivates variable selection procedures in statistical post-processing.

A random forest is generally seen as a method that deals rather well with high dimensional covariates. This property comes from the fact that in the tree fitting algorithm, a random forest chooses the split variables and split points in a greedy way, based on a certain criterion, e.g. the variance. This is often rather effective in the beginning of the tree fitting as many observations are split, but deep down in the tree there are fewer observations which makes the splitting criterion subject to higher variances. Therefore global variable selection methods are considered in the literature to improve statistical efficiency and interpretation of the random forest model.

Variable selection in random forests is mainly done in terms of two types of importance measures. The first type calculates the decrease in impurity of a split made in a tree. In [13] consistency of these measures is shown on fully randomized trees. But in practice in a random forest setting these impurity measures are shown to exhibit biases [17]. The second type is the permutation measure introduced in [2]. This measure computes how much the predictive performance decreases by randomly permuting one single predictor, which breaks the relation between response and the predictor. A popular approach is to perform a backward selection based on the permutation measures, where the model with the best predictive performance is chosen, see e.g. [5,6,9].

Correlation between predictors has a large effect on the permutation importance scores. An initial approach of dealing with this is to consider conditional importance scores, [16]. This has the downside that in some way the conditioning variable has to be chosen. A more precise analysis of the effect of correlation on permutation measures is done in [9], where they conclude that a backward selection is better able to handle correlation between predictors than other strategies incorporating variable importance measures. We show in our simulation study that although the correct variables are often selected by the backward selection, there is no control on the rate of selected noise variables, i.e. the false positives.

In this paper, we propose a new method of selecting variables with random forests. The developed method aims to improve the probabilistic forecasts. Let Y∈R denote the response variable and X denote the covariate vector. Given an observed covariate x, a deterministic forecast provides a single value, for instance E(Y∣X=x) as the popultation prediction, whereas a probabilistic forecast offers a conditional distribution of *Y* given X=x as the prediction. The advantage of a probabilistic forecast is that it directly communicates forecast uncertainties that enable the users to make better decisions [3,15]. A commonly used measure of performance for probabilistic forecasts of a scalar variable is the so-called continuous ranked probability score [7]. In our variable selection procedure, we will use this score as the loss function (cf. (1)) to select variables that are informative for the entire conditional distribution instead of just for the conditional mean. The procedure estimates the predictive risk based on the so-called out-of-bag samples (cf. Section 3.2), which is similar to leave-one-out cross validation. At each step, the variable that leads to the smallest CRPS risk is selected and the selection stops if the decrease in CRPS risk is not significantly different from zero. We show by a detailed simulation study that our method controls the false positive rate much better than the backward selection method introduced in [9], even in the presence of high correlations.

The outline of the paper is as follows. In Section 2, we give a detailed description of the mathematical set-up of the variable selection procedure. Then in Section 3, we give a small introduction to random forests and show how the variable selection can be applied to the random forest set-up. A comparison on correlated covariates is given in Section 4 where we compare with an approach selecting variables in a backward selection based on permutation measures. In Section 5, we apply the method to a practical example of post-processing maximum temperature forecasts and compare it to a standard method in post-processing. Finally, we end with a discussion in Section 6.

## 2. Forward selection

In this section, we describe the mathematical set-up of our forward variable selection method. We provide the intuition of the procedure together with some theoretical motivation of the method in the case of independent covariates.

For now, we consider a pair of random observations (X,Y), where the response variable Y∈R and the covariate vector $X\in R^{d}$. Let J⊂{1,…,d} denote a set of indices corresponding to the entries of the covariate vector X and $X^{J}$ denote the vector with the entries from X corresponding to *J*. Let $F_{Y|X^{J}}$ denote the conditional distribution function of *Y* given $X^{J}$. In this section, we work from the population perspective and consider the exact distribution function $F_{Y|X^{J}}$ as a forecast of *Y*. The next section will be concerned with the estimation of the conditional distributions using random forests.

As motivated in Section 1, we are interested in a probabilistic forecast. We use the continuous rank probability score (CRPS) to measure the distance between the degenerate cdf on {Y} and $F_{Y|X^{J}}$:

(1) $$\mathrm{CRPS}(Y,F_{Y|X^{J}}):=\int_{-\infty }^{\infty }{\left(I(Y\leq z)-F_{Y|X^{J}}(z|X^{J})\right)}^{2}\;\text{d}z.$$

The discrete rank probability score was first introduced in [4]. The application of CRPS was limited due to a lack of tractability of the integral. During the past decades, the CRPS has attracted renewed interest, in particular in the community of meteorology and atmosphere science; see [10,22] among others. As a loss function, the CRPS has two attractive properties. First, it is a proper scoring rule for a large class of distribution functions; see Section 4.2 in [7]. Second, there exists an equivalent expression of the CRPS in terms of the conditional quantile function. Denote the conditional quantile function by $Q_{Y|X^{J}}(\tau |X^{J})$, where τ∈(0,1) is a probability level. Then the CRPS can be expressed as

(2) $$\mathrm{CRPS}(Y,F_{Y|X^{J}})=2\int_{0}^{1}\rho_{\tau }(Y-Q_{Y|X^{J}}(\tau |X^{J}))\;\text{d}\tau =:\mathrm{CRPS}(Y,Q_{Y|X^{J}}),$$

where $\rho_{\tau }(u)=u(\tau -I(u<0))$ the quantile check function. This equivalent expression of CRPS plays an import role when it comes to the risk estimation. It enables us to evaluate the loss by estimating the conditional quantile via a random forest, detailed in the next section. The equivalence of these two expressions is shown in the appendix. In addition, in Theorem 2.1 below we show that with CRPS as the loss, the procedure is able to retrieve the informative variables correctly.

Corresponding to the CRPS loss, we can now define a risk for the subset of variables corresponding to *J* as

(3) $$R(J):=E[\mathrm{CRPS}(Y,F_{Y|X^{J}})].$$

In our approach an ideal variable selection procedure selects the set of variables corresponding to *J* that minimizes this risk. Define $m_{R}=\min_{J\subset \{1,\ldots ,d\}}R(J)$. Then, an optimal set of variables denoted by $X^{J^{*}}$ is such that

(4) $$R(J^{*})=m_{R}\;\mathrm{and}\;|J^{*}|=\min\{|J|:R(J)=m_{R}\}$$

where |J| denotes the cardinality of *J*. The goal is to identify the smallest model that reaches an optimal risk. This is desirable when it comes to estimating the conditional distribution of *Y*. It is important to note that $J^{*}$ is not necessarily unique. For example two collinear covariates $X_{1}$ and $X_{2}$ both contain the same information on *Y*, then including any of the two covariates would result in the same expected loss.

In order to obtain $J^{*}$, one could evaluate R(J) for all $2^{d}$ possible sets, which is often computationally infeasible. Instead we propose a forward variable selection approach as follows. We construct a sequence of length *d* + 1 of nested sets $J_{j}$ for j=0,…,d where $J_{0}=\emptyset$ and

(5) $$J_{j}=J_{j-1}\cup \left\{{\arg\;\min}_{q\notin J_{j-1}}R\left(J_{j-1}\cup \{q\}\right)\right\}.$$

Our proposed forward selection procedure selects an optimal set $J^{o}$ such that it is the smallest set attaining the minimum risk among $J_{j}$, j=0,…d. More precisely,

(6) $$J^{o}=J_{\min\{j:R(J_{j})=\min_{0\leq i\leq d}R(J_{i})\}}.$$

In the theorem below we show that under the assumption of independent covariates, the sets $J^{o}$ and $J^{*}$ coincide.

Theorem 2.1 Let $X_{1},\ldots ,X_{d}$ and *ϵ* be independent random variables. Let $h:R^{|J^{*}|+1}\to R$ be a real valued measurable function and define $Y=h(X^{J^{*}},\epsilon )$, where $J^{*}\subseteq \{1,\ldots ,d\}$. Assume that $E[Y^{2}]<\infty$, and for any $I⊊J\subseteq J^{*}$, there exists a set S⊆R with positive Lebesgue measure such that $E[I(Y\leq z)|X^{J}]$ is not $\sigma (X^{I})$ measurable for all z∈S. Then $J^{*}$ is the unique subset of {1,…,d} satisfying (4), and $J^{0}=J^{*}$.

Proof. Let (Ω,A,μ) denote the probability space supporting $X_{1},\ldots ,X_{d}$ and *ϵ*. Define the standard inner product on $L^{2}(\Omega ,A,\mu )$ by $\left(Z_{1},Z_{2})=E(Z_{1}Z_{2}\right)$, for any random variables $Z_{1}$ and $Z_{2}$ on (Ω,A,μ). Then $L^{2}(\Omega ,\sigma (X_{1},\ldots ,X_{d},\epsilon ),\mu )$ becomes a Hilbert space, where the conditional expectation $E(Z|X^{J})$ is the orthogonal projection of *Z* onto the closed linear subspace $L^{2}(\Omega ,\sigma (X^{J}),\mu )$. Now we have

$$\begin{array}{rl} R(J) & =\int_{-\infty }^{\infty }E\left[{\left(I(Y\leq z)-F_{Y|X^{J}}(z|X^{J})\right)}^{2}\right]\;\text{d}z\\ & =\int_{-\infty }^{\infty }E\left[{\left(I(Y\leq z)-E[I(Y\leq z)|X^{J}]\right)}^{2}\right]\;\text{d}z\\ & =:\int_{-\infty }^{\infty }g_{J}(z)\;\text{d}z. \end{array}$$

As the conditional expectation equals the orthogonal projection, for any z∈R,

(7) $$g_{J}(z)=\min_{G\in \sigma (X^{J})}E[(I(Y\leq z)-G{)}^{2}].$$

Therefore, for any z∈R, if $J_{1}\subset J_{2}$, we have

(8) $$g_{J_{1}}(z)\geq g_{J_{2}}(z).$$

This implies that $R(J_{1})\geq R(J_{2})$.

Next, note that if $J_{2}=J_{1}\cup \{j\}$ and $j\notin J^{*}$, then for any z∈R,

(9) $$g_{J_{1}}(z)=g_{J_{2}}(z).$$

This is because $E[I(Y\leq z)|X^{J_{2}}]=E[I(Y\leq z)|X^{J_{1}}]$ by the independence of *Y* and $X_{j}$. In this case $R(J_{1})=R(J_{2})$.

Finally, we show that if $J_{2}=J_{1}\cup \{j\}$, where $j\in J^{*}$ then $R(J_{1})>R(J_{2})$. We prove by contradiction. If not, then $R(J_{1})=R(J_{2})$, which means in view of (8) that $g_{J_{1}}(z)=g_{J_{2}}(z)$, for all z∈R∖C, where *C* has zero Lebesgue measure.

From here we denote I(Y≤z) by $I_{z}$ to simplify notation. Expanding the squares and using the tower property of conditional expectation we see that

$$\begin{array}{rl} g_{J_{1}}(z)-g_{J_{2}}(z) & =E\left[{\left(I_{z}-E[I_{z}|X^{J_{1}}]\right)}^{2}-{\left(I_{z}-E[I_{z}|X^{J_{2}}]\right)}^{2}\right]\\ & =E\left[-2I_{z}E[I_{z}|X^{J_{1}}]+E[I_{z}|X^{J_{1}}{]}^{2}+2I_{z}E[I_{z}|X^{J_{2}}]-E[I_{z}|X^{J_{2}}{]}^{2}\right]\\ & =E\left[E\left[-2I_{z}E[I_{z}|X^{J_{1}}]+E[I_{z}|X^{J_{1}}{]}^{2}+2I_{z}E[I_{z}|X^{J_{2}}]-E[I_{z}|X^{J_{2}}{]}^{2}\right|X^{J_{2}}]\right]\\ & =E\left[(E[I_{z}|X^{J_{1}}]-E[I_{z}|X^{J_{2}}]{)}^{2}\right]=0. \end{array}$$

From this we conclude that $E[I_{z}|X^{J_{1}}]=E[I_{z}|X^{J_{2}}]$ for all z∈R∖C. This implies that $E[I_{z}|X^{J_{2}}]$ is $\sigma (X_{J_{1}})$ measurable which contradicts our assumption, hence $R(J_{1})>R(J_{2})$.

We can now observe that the forward sets are built by adding variables from $J^{*}$ until all variables of $J^{*}$ have been added, therefore $J^{0}=J^{*}$.

Remark 2.1 Apart from measurability, we do not impose constraints on the function *h* and the noise term *ϵ*. It allows a large class of models including additive models, models with interaction terms, etc. When it comes to the estimation, we will use a random forest which is a model-free statistical learning method.

Remark 2.2 The assumption that $E[I(Y\leq z)|X^{J}]$ is not $\sigma (X^{I})$-measurable for any $I⊊J\subseteq J^{*}$, is needed to prove the uniqueness of $J^{*}$. As we know that $R(J^{*})=R(J^{*}\cup \{j\})$ for $j\notin J^{*}$, there are many sets, which have minimal risk in population sense. The assumption essentially ensures that $J^{*}$ does contain only indices *j* such that the function *h* is not constant for $x_{j}$ almost everywhere with respect of the distribution of *X*.

## 3. Forward selection using random forests

We use a random forest to estimate the conditional distribution function $F_{Y|X^{J}}$ and the risk. Now, we make a little excursion to explain the random forest algorithm. We follow the tree construction algorithm proposed in [20] and the extension for quantile estimation from [1]. We choose this approach because it is the only approach that makes splits based on a quantile criterion, additionally in [1] asymptotic normality for the quantile estimates is established.

### 3.1. Intermezzo: random forests

Denote the data set by $\left(X_{1},Y_{1}),\ldots ,(X_{n},Y_{n}\right)$. A random forest is defined as a collection of trees. Each tree *T* is obtained by recursively splitting a set of observations by making axis-aligned splits in the covariate space, meaning a split is made on a single covariate value at a time. As a result, every tree induces a partitioning of the covariate space in possibly semi-infinite hyper rectangles. Denote the conditional quantile function by $Q_{Y|X}(\tau |\cdot )$, where τ∈(0,1) denotes a probability level. In this section, we focus on fitting a forest in order to estimate the function $Q_{Y|X}(\tau |\cdot )$. The estimation procedure for $Q_{Y|X^{J}}(\tau |X^{J})$ works exactly the same by fitting a forest-based on $\left\{(X_{1}^{J},Y_{1}),\ldots ,(X_{n}^{J},Y_{n})\right\}$.

Recurrent splits are made starting with parent node *P*, a node in the current partition, creating two child nodes $C_{1}$ and $C_{2}$, such that $P=C_{1}\cup C_{2}$ and $C_{1}\cap C_{2}=\emptyset$. This split should be informative with respect to $Q_{Y|X}(\tau |\cdot )$ and is chosen to maximize,

(10) $$e(C_{1},C_{2})=\frac{n_{C_{1}}n_{C_{2}}}{n_{P}}(Q_{Y|X}(\tau |X\in C_{1})-Q_{Y|X}(\tau |X\in C_{2}){)}^{2},$$

where $n_{P}$, $n_{C_{1}}$, $n_{C_{2}}$ are the number of observations $X_{i}$ in each node. In practice this makes the algorithm very slow as it requires the computation of two quantiles for each possible split. Instead in [1] a relabeling step is proposed and defined as $I(Y_{i}>Q_{Y|X}(\tau |X\in P))$ for the *τ* quantile. Now a standard regression split, as used in a standard random forest [2], is made on the labels. This means to maximize the squared difference between the average label in both child nodes.

The trees fitted in [1,20] are called honest trees and are slightly different from the standard structure of tree fitting. A tree is fit by first sub-sampling a set of indices from {1,…,n} of size *s*<<*n* and then randomly splitting this sub-sample in two sets I and J both of size *s*/2. Recursive splits of $R^{d}$ are then made based on criterion (10), with data points $(X_{i},Y_{i}):i\in I$. The tree becomes honest by removing all the data points indexed by set I and using only the data points indexed by set J for estimation of $Q_{Y|X}(\tau |x)$ for new observations X.

A random forest is then obtained by fitting *B* trees. Denote by $l_{b}(X)$ the leaf node of tree *b* in which X falls. Then for 1≤i≤n, the weight for $\left(X_{i},Y_{i}\right)$ induced by the *b*th tree is given by,

(11) $$w_{i,b}(X)=\frac{I(i\in J\;\&\;X_{i}\in l_{b}(X))}{\sum_{j\in J}I(X_{j}\in l_{b}(X))},$$

where $\frac{0}{0}=0$. The forest weights are obtained by averaging the tree weights over the *B* trees, $w_{i}(x)=\frac{1}{B}\sum_{b=1}^{B}w_{i,b}(X)$. An estimate of ${\hat{Q}}_{Y|X}$ is then given by the locally weighted estimated quantile,

(12) $${\hat{Q}}_{Y|X}(\tau |x)={\arg\;\min}_{\theta }\sum_{i=1}^{n}w_{i}(x)\rho_{\tau }(Y_{i}-\theta ),$$

with $\rho_{\tau }(u)=u(\tau -I(u<0))$ the quantile check function. Note that the structure is similar to kernel regression, but instead of a deterministic kernel with bandwidth *h* the weights are determined by the data via the forest. Random forests are sometimes called adaptive nearest neighbor estimators for this reason. The tree weights $w_{i}$ can also be used to obtain the estimator of conditional distribution function:

(13) $${\hat{F}}_{Y|X}(y|x)=\sum_{i=1}^{n}w_{i}(x)I(Y_{i}\leq y).$$

In the variable selection procedure we aim to select variables that are predictive for the conditional distribution. Therefore, instead of building random forests with respect to a single *τ* quantile, consider a sequence of quantiles $0<\tau_{1},\ldots ,\tau_{K}<1$. This needs a different type of relabeling than for a single quantile as explained above. They define the relabeling then by,

$$Z_{i}=\sum_{k=1}^{K}I(Y_{i}\leq {\hat{Q}}_{Y|X}(\tau_{k}|X\in P)).$$

The best split is then chosen to maximize the following multi-class classification rule:

$$\hat{e}(C_{1},C_{2})=\frac{\sum_{k=1}^{K}{\left[\sum_{X_{i}\in C_{1}}I(Z_{i}=k)\right]}^{2}}{n_{C_{1}}}+\frac{\sum_{k=1}^{K}{\left[\sum_{X_{i}\in C_{2}}I(Z_{i}=k)\right]}^{2}}{n_{C_{2}}}.$$

### 3.2. Estimation of predictive loss

The main quantity in the theoretical framework from Section 2 is the CRPS risk. Replacing the theoretical conditional quantile function by its estimator in (2), we obtain the following targeted loss in the estimation context:

(14) $$\mathrm{CRPS}(Y,{\hat{Q}}_{Y|X^{J}})=2\int_{0}^{1}\rho_{\tau }(Y-{\hat{Q}}_{Y|X^{J}}(\tau |X^{J}))\;\text{d}\tau .$$

Here we denote by ${\hat{Q}}_{Y|X^{J}}$ the random forest estimator of the conditional quantile function with respect to the dataset $\left\{(X_{1}^{J},Y_{1}),\ldots ,(X_{n}^{J},Y_{n})\right\}$ and with two arguments, a probability level *τ* and the covariate vector $X^{J}$.

A naive way to estimate the expected loss (that is the expectation of (14)), would be considering

$$\frac{2}{n}\sum_{i=1}^{n}\int_{0}^{1}\rho_{\tau }(Y_{i}-{\hat{Q}}_{Y|X^{J}}(\tau |X_{i}^{J}))\;\text{d}\tau .$$

However, this would lead to over-fitting because the training set (data for estimating $Q_{Y|X^{J}}$) is the same as the testing set (data for estimating the expectation). This problem can be circumvented by using so called out-of-bag samples as test set.

The out-of-bag samples for the *b*th tree are defined as the samples that are not used for generating the tree. For each observation $\left(X_{i}^{J},Y_{i}\right)$, a sub forest $F_{i}$ is defined by $F_{i}=\{T_{b}:i\notin (I_{b}\cup J_{b})\}$. Namely, this sub forest consists of trees for which $\left(X_{i}^{J},Y_{i}\right)$ is out-of-bag. Observe that the number of trees in $F_{i}$ is random and hence not necessarily the same for all *i*. The expected number of trees for each sub forest is $B(1-\frac{s}{n})$.

We use the sub forest $F_{i}$ to estimate the conditional quantile function and denote it with ${\hat{Q}}_{Y|X}^{F_{i}}(\tau |X^{J})$. Since the trees in sub forest $F_{i}$ do not use observation $\left(X_{i}^{J},Y_{i}\right)$, we use this quantile estimator to evaluate the CRPS loss for $\left(X_{i}^{J},Y_{i}\right)$. Doing this for all observations, we obtain the estimated CRPS risk given by,

(15) $$\hat{R}(J):=\frac{2}{n}\sum_{i=1}^{n}\int_{0}^{1}\rho_{\tau }(Y_{i}-{\hat{Q}}_{Y|X^{J}}^{F_{i}}(\tau |X_{i}^{J}))\;\text{d}\tau .$$

In the sequel, we write ${\hat{Q}}_{Y|X^{J}}^{F_{i}}(\tau |X_{i}^{J})={\hat{Q}}^{F_{i}}(\tau |X_{i}^{J})$ for simplicity.

This out-of-bag procedure for estimating risk has similarities with leave-one-out cross validation. For validating the quantile of the *i*th observations we use all trees which do not use the *i*th observation. The difference is that sub forests have in expectation the same size, but not exactly. Computationally the out-of-bag sample approach is also much faster compared to leave-one-out cross validation. Note that a tree has *n*−*s* out-of-bag samples and hence the tree is used in *n*−*s* sub-forests. On the other hand leave one out cross validation does not reuse trees and estimates a new forest for each element in the summation of (15).

### 3.3. One step forward

The forward variable selection sequentially adds variables such that the predictive loss is minimized. We here explain how each step is performed. Let *d* denote the total number of covariates and $d^{\prime }$ denote the number of signal variables, that is $d^{\prime }=|J^{*}|$. In order for the estimation procedure to work, we need a sparsity assumption: $d^{\prime }<<d$.

Recall that for an index set *J*, the estimated risk $\hat{R}(J)$ is given by (15). Suppose that we have selected the first *j*−1 variables with indices in ${\hat{J}}_{j-1}$. Then the *j*th variable $X_{{\hat{i}}_{j}}$ is selected based on

(16) $${\hat{i}}_{j}={\arg\;\min}_{q\notin {\hat{J}}_{j-1}}\hat{R}({\hat{J}}_{j-1}\cup \{q\}).$$

and ${\hat{J}}_{j}={\hat{J}}_{j-1}\cup \{{\hat{i}}_{j}\}$. The procedure of a single step forward is detailed in Algorithm 1.


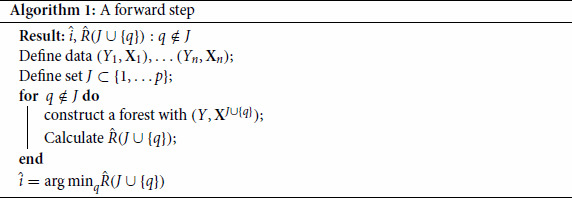


### 3.4. Stopping criterion

Motivated by the result in Theorem 2.1, we stop selecting variables when there is no further decrease in CRPS risk. From the proof of Theorem 2.1, adding variables that are not in $J^{*}$ does not have an effect on the CRPS risk. In practice, where we are working with finite samples, additional covariates decrease in fact the statistical efficiency of the random forest which leads to higher CRPS values. Because of the random component in the forest procedure, different forests will have different risk. In general this can be avoided by fitting an enormous number of trees to reduce the random component, but in practice this is infeasible due to the computational load. Instead we make use of the randomness to design a stopping criterion. Ideally, the procedure stops at *j*th step if $R({\hat{J}}_{j-1})-R({\hat{J}}_{j})=0$ and continues if the difference is positive. However, the difference is unknown and we estimate it via the already fitted forests at *j*th and (j+1)th steps. More precisely, we estimate this difference by $\hat{R}({\hat{J}}_{j-1}\cup \{q\})-\hat{R}({\hat{J}}_{j}\cup \{q\})$, where $q\notin {\hat{J}}_{j}$. Note that $\hat{R}({\hat{J}}_{j-1}\cup \{q\})$ is computed at the *j*th step for identifying ${\hat{i}}_{j}$ and $\hat{R}({\hat{J}}_{j}\cup \{q\})$ at the (j+1)th step for identifying ${\hat{i}}_{j+1}$. So, this estimation does *not* require any extra forest fitting.

Now suppose that the variable added at *j*th step is a noise variable. Then for any *q*, $R({\hat{J}}_{j-1}\cup \{q\})-R({\hat{J}}_{j}\cup \{q\})=0$ and the estimate $\hat{R}({\hat{J}}_{j-1}\cup \{q\})-\hat{R}({\hat{J}}_{j}\cup \{q\})$ fluctuates around zero due to the randomness in the estimation. Therefore,

$$I(\hat{R}({\hat{J}}_{j-1}\cup \{q\})-\hat{R}({\hat{J}}_{j}\cup \{q\})>0)\overset{\mathrm{asym}.}{∼}\mathrm{Bern}(0.5).$$

Combining all the information for $q\notin {\hat{J}}_{j}$, we define

(17) $$W_{j}:=\sum_{q\notin {\hat{J}}_{j}}I(\hat{R}({\hat{J}}_{j-1}\cup \{q\})-\hat{R}({\hat{J}}_{j}\cup \{q\})>0).$$

Then approximately $W_{j}∼\text{Bin}(M_{j},0.5)$, where $M_{j}=d-|{\hat{J}}_{j}|$ under the assumption that the *j*th selected variable is a noise variable. The procedure stops at *j*th step if the following stopping criterion is met: $W_{j}<C_{1-\alpha }^{j},$ where $C_{1-\alpha }^{j}$ is the 1−α quantile of $\text{Bin}(M_{j},0.5)$ and α∈(0,1) is a pre-specified level. The mathematical motivation for the stopping criterion is provided in the appendix.

In practice, the integration in (15) is numerically approximated. Let $\tau_{t}=\frac{t}{k+1},t=1,\ldots ,k$, where *k* is a pre-specified integer. The estimated risk $\hat{R}(J)$ in (15) is approximated by

(18) $$\hat{R}(J)=\frac{2}{k}\sum_{t=1}^{k}\rho_{\tau_{t}}(Y_{i}-{\hat{Q}}_{Y|X}(\tau_{t}|X)).$$

The complete procedure is given in Algorithm 2.

Remark 3.1 This stopping criterion works well when $M_{j}$ (number of variables not been selected yet) is sufficiently large. This is more or less assured by our sparsity assumption of $d^{\prime }<<d$. In other words, this algorithm assumes that there are sufficiently amount of noise variables. This is also the situation when variable selection is interesting.

For the situation when there are only a few or no noise variables, we need some adjustment in the algorithm. The algorithm stays the same until $M_{j}$ becomes small, that is, most variables are selected. Then stopping criterion in (17) will automatically stop with selecting new variables. Because for a small $M_{j}$, one has $C_{1-\alpha }^{j}=M_{j}$ due to the discreteness of a binomial distribution and the stopping criterion $W_{j}<C_{1-\alpha }^{j}$ is met. For instance, if $M_{j}=4$ then $C_{0.95}^{j}=4$. To adjust the algorithm to accommodate this issue, we suggest to estimate the risk differently. For a sufficiently large *L*, we fit 2L random forests to estimate the risk $R({\hat{J}}_{j-1})$ and $R({\hat{J}}_{j})$, each with *L* forest respectively. The adjusted stopping criterion is defined as

$${\tilde{W}}_{j}:=\sum_{l=1}^{L}I({\hat{R}}_{l}({\hat{J}}_{j-1})-{\hat{R}}_{l}({\hat{J}}_{j})>0),$$

where ${\hat{R}}_{l}({\hat{J}}_{j-1})$ is the estimate of $R({\hat{J}}_{j-1})$ based *l*th random forest. The procedure stops if ${\tilde{W}}_{j}<{\tilde{C}}_{1-\alpha }$, where ${\tilde{C}}_{1-\alpha }$ is the 1−α quantile of Bin(L,0.5). Note that this is computationally much more demanding due to the fact that 2L more random forest models are computed at each step.

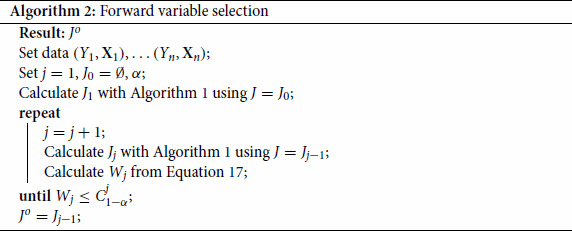


## 4. Comparison based on simulation

In this section we assess the performance of our variable selection procedure for correlated explanatory variables. We will compare with the backward selection based on a permutation measure with a mean squared error criterion proposed in [9]; details of the method are stated later in this section. We compare with this method as it is currently the only method that deals with selection with correlated predictors for random forests and we will refer to it as the backMSE method. For the comparison we simulate data from the following model,

(19) Y=μ(X)+σ(X)ϵ,

where *ϵ* follows a standard normal distribution and independent of this, $X\in R^{25}$ follows a multivariate normal distribution. For the covariance structure of X we split up the covariates into blocks $I_{l}=\{(l-1)*5+1,\ldots ,(l-1)*5+5\}$ for l=1,…,5. The covariance matrix of X is then given by,

(20) $$\text{Cov}(X_{j},X_{i})=\left\{ \begin{array}{ll} 1, & \text{if}\;i=j;\\ \rho , & \text{if}\;i,j\in I_{l}\;\text{for the same}\;l;\\ \eta , & \text{ otherwise.} \end{array}\right.$$

Observe that the value of *ρ* controls the correlation strength within each block and *η* controls the strength between blocks. We generate data from four simulation models with sample size n∈{500,1000,2500}. In the first three models, the covariates are simulated with a block independent structure where signal variables are independent of each other. Precisely for Models 1-3, we set ρ∈{0,0.4,0.8} and η=0. The *μ* and *σ* for each model are specified below. The sparsity assumption made in Section 3.3 is satisfied within these models as *d* = 25 and $d^{\prime }=3$.

Model 1:
$\mu (X)=X_{1}+\frac{X_{6}}{2}+\frac{X_{11}}{4}$ and σ(X)=1.
Model 2:
$\mu (X)=X_{1}$ and $\sigma (X)=\exp(\frac{X_{6}}{2}+\frac{X_{11}}{3})$.
Model 3:
$\mu (X)=\left\{ \begin{array}{l} X_{6}^{2},\;\text{if}\;X_{1}\geq 0,\\ -X_{6},\;\text{if}\;X_{1}<0, \end{array}\right.$ and $\sigma (X)=\left\{ \begin{array}{l} 2,\;\text{if}\;X_{11}\geq 0,\\ 1,\;\text{if}\;X_{11}<0. \end{array}\right.$

For the first three models, with different values of *ρ* we investigate the effect of different correlation strengths between the noise variables and the signal variables on the selection procedure. The difference between the three models is in how the signal variables influence the response. In the first model *Y* only depends on covariates through its mean function. In the second model the dependence is both on the main and the variance. The third model considers discontinuous covariate dependence on both mean and variance.

We consider a fourth model which introduces correlation cross blocks, so the signal variables are dependent now and the covariance matrix of predictors is no longer block diagonal. The dependence between *Y* and the signal variables is the same as Model 1.

Model 4:
$\mu (X)=X_{1}+\frac{X_{6}}{2}+\frac{X_{11}}{4}$ and σ(X)=1, where ρ=0.8 and η∈{0.2,0.4,0.6}.

The backMSE method evaluates the relevance of a covariate by its permutation importance measure, which is defined as

$$I(X_{j})=E\left[(Y-E(Y|X_{\left(j\right)}){)}^{2}\right]-E\left[(Y-E(Y|X){)}^{2}\right],$$

where $X_{\left(j\right)}=(X_{1},\ldots ,X_{j}^{\prime },\ldots ,X_{d})$ such that $X_{j}^{\prime }{=}^{d}X_{j}$ and $X_{j}^{\prime }$ is independent of *Y* and of the other covariates. A large score of $I(X_{j})$ indicates that covariate $X_{j}$ is important. The method randomly permutes the values of $X_{j}$ to mimic a random sample of $X_{j}^{\prime }$. An estimator of $I(X_{j})$ using out of bag samples is given in (2.1) in [9].

In [9] it is shown that the order of the permutation importance measures can not be naturally interpreted in the presence of correlation between the covariates, as variables that are correlated share their importance. As a result, the importance of the important variables is lower than it should be. The backMSE deals with this problem by iteratively removing the least important variable and refitting the model and calculating the importance scores. This process is repeated until no variables are left. The optimal model is then chosen as the model that minimizes the out-of-bag mean squared error. Why this works is easily seen with two highly correlated informative variables. Initially they do not seem important because they share their importance, but by removing one the importance is not shared any more. The left over variable shows the true importance and will therefore be in the selected set.

It is recommended in [9] to compute several forests and take averages to stabilize the variable importance scores and the error estimates. We compute for each step 20 forests where each forest contains 2000 trees. For this method, we follow the standard forest algorithm from [2], fitting trees based on bootstrap samples of size *n*, *mtry* is set to the default value for regression *p*/3 and taking a minimum leaf size of 5.

For our method we also take fixed parameters with sub-sampling fraction s=0.5, a minimum node size of 1, *mtry* = *p* and 1000 trees. We have tested the influence of these tuning parameters on several simulation models and the results are rather robust to different choices. Our selection model adds variables one at a time and stops when additional variables do not increase performance. As the model is therefore often small it makes sense to not over randomize by setting *mtry* to smaller than *p*. We advise to choose a small *s* for large datasets in order to reduce computation time.

**Figure 1.**
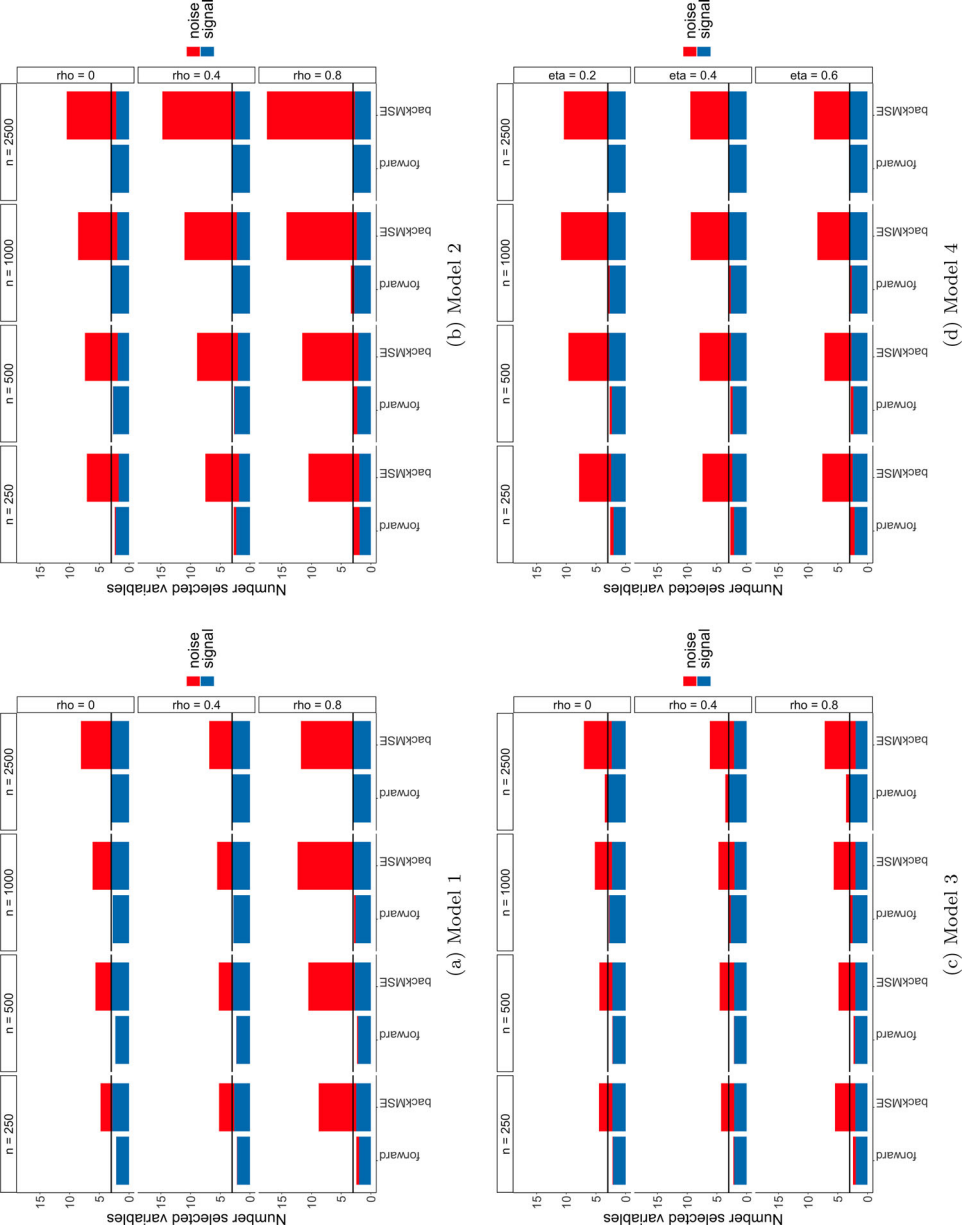
Average number of selected variables over 100 simulations. Total number of variables is the height of the bar blue for signals variables and red for noise variables. Bars from left to right correspond to the sample sizes and top to bottom corresponds to different levels of correlation, except for Model 4 where top to bottom corresponds to minimum correlation strength between blocks. The horizontal line coincides the total number of signal variables in the model.

For each model we simulate 100 data sets. The results are summarized in Figure 1. We show for each model, sample size and covariate dependence structure the average number of correctly selected signal variables in blue, the average number of incorrectly selected noise variables in red and the total number of signal variables with the black line. From this the average number of missed signal variables is visualized as the difference between the black line and the height of the blue bar.

For the first model we see that the backMSE method retrieves more signal variables than the forward selection for low sample sizes and that as the sample size grows the forward variable selection also recovers all signal variables. A large difference is seen in the number of noise variables that are selected. The forward selection performs much better in this than the backMSE, which systematically selects noise variables and tends to even select more as the sample size increases. This phenomenon is also visible for Models 2 and 3 as seen in Figure 1(b, c). For these two models where the variance is dependent on covariates, the CRPS criterion clearly has an edge over the backMSE that selects variables based on the mean squared error and therefore has a hard time selecting these variables. For Model 4 similar results are visible to the Model 1 with correlation strength ρ=0.8, indicating that increased correlation between signal variables does not affect the overall quality of the result.

The reason why the backMSE selects many noise variables is two-fold. First the backMSE method selects the optimal set based on a predictive mean squared error criterion. This approach does not account for the inherent variable selection within the random forest, where at each node the split that reduces the variance the most is chosen. As a result the random forest is able to ignore noise variables partially. In practice this means that in an out-of-bag performance measure the addition of a single noise variable cannot be detected. Therefore the variables that are selected will not be the smallest set, but instead a set with maximum number of noise variables maintain the lowest performance. Secondly, the backMSE does not adequately deal with the correlation. For example in Model 1 with ρ=0.8 all variables $X_{1},\ldots ,X_{5}$ have higher variable importance compared to $X_{6}$, which means that if $X_{6}$ is in the model, so are $X_{2},\ldots ,X_{5}$.

Thanks to our testing approach, a small number of noise variables is selected with the forward selection. Using the randomness induced by the random forest, our testing procedure selects a variable that leads to a significant reduction of the predictive loss. The significance level naturally controls the number of selected noise variables by the nature of the testing procedure. We have set the significance level to 5% for all simulations in the paper.

### 4.1. Variable selection in the setting *d*>*n*

We end this section with a simulation in a situation where *d*>*n*, i.e. when there are more variables than observations, and show that our method is able to deal with these high dimensional problems. The dimension of the covariate space is set to *d* = 150 and sample size *n* = 100. The dependence between the covariates has a similar block independence structure as for Model 1, but the number of blocks is now 30. Dependence between the covariates and the response is defined by (19) with functions,

$$\begin{array}{rl} & \mu (X)=X_{1}+X_{6}+X_{11}\\ & \sigma (X)=1. \end{array}$$

**Figure 2.**
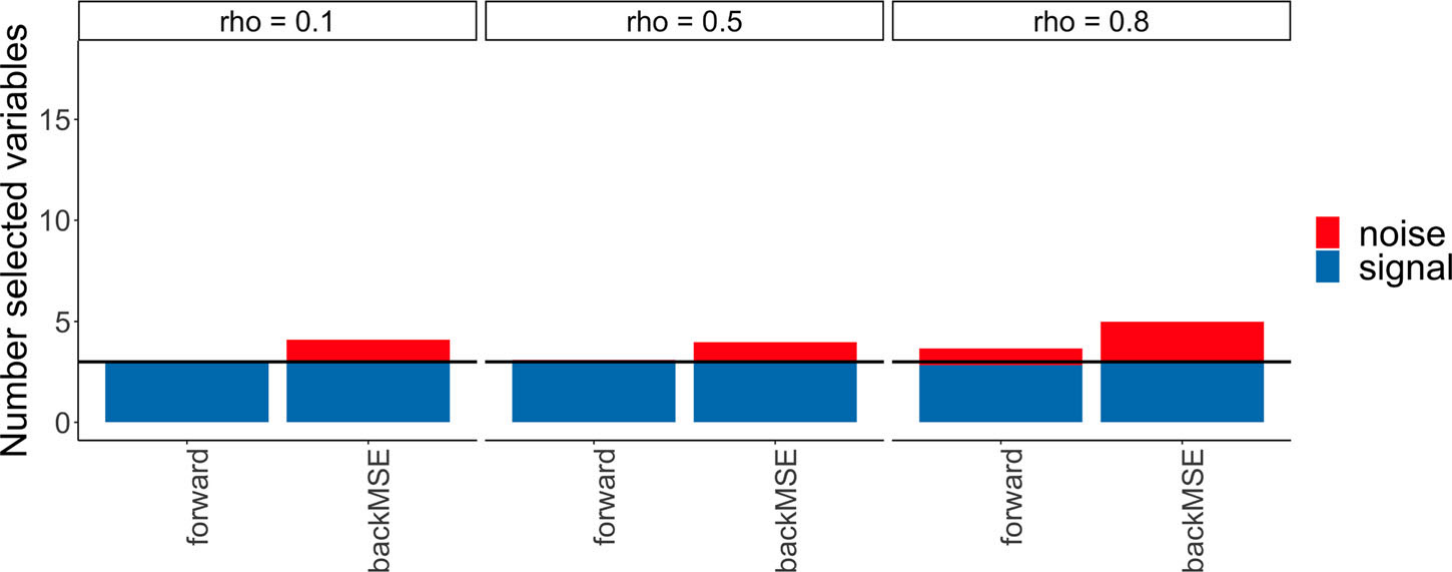
Average number of selected variables over 100 simulations for *n* = 100 and *d* = 150. The blue bars correspond to the signal variable the red bars to the noise variable. The covariate dependence structure from left to right changes with the indicated correlation strength (see label). The horizontal black line coincides with the total number of signal variables in the model.

Note that also here the sparsity assumption is satisfied with *d* = 150 and $d^{\prime }=3$. The experiment is repeated for 100 times and the results for our forward method and the backMSE method are displayed in Figure 2.

It can be observed that our method is able to select all signal variables all of the time, only when correlation strength is 0.8, more variables are selected. The backward method again systematically selects more noise variables for all correlation strengths, which is consistent with the results observed in the setting with *d*<*n*.

## 5. Post-Processing maximum temperature forecasts

**Figure 3.**
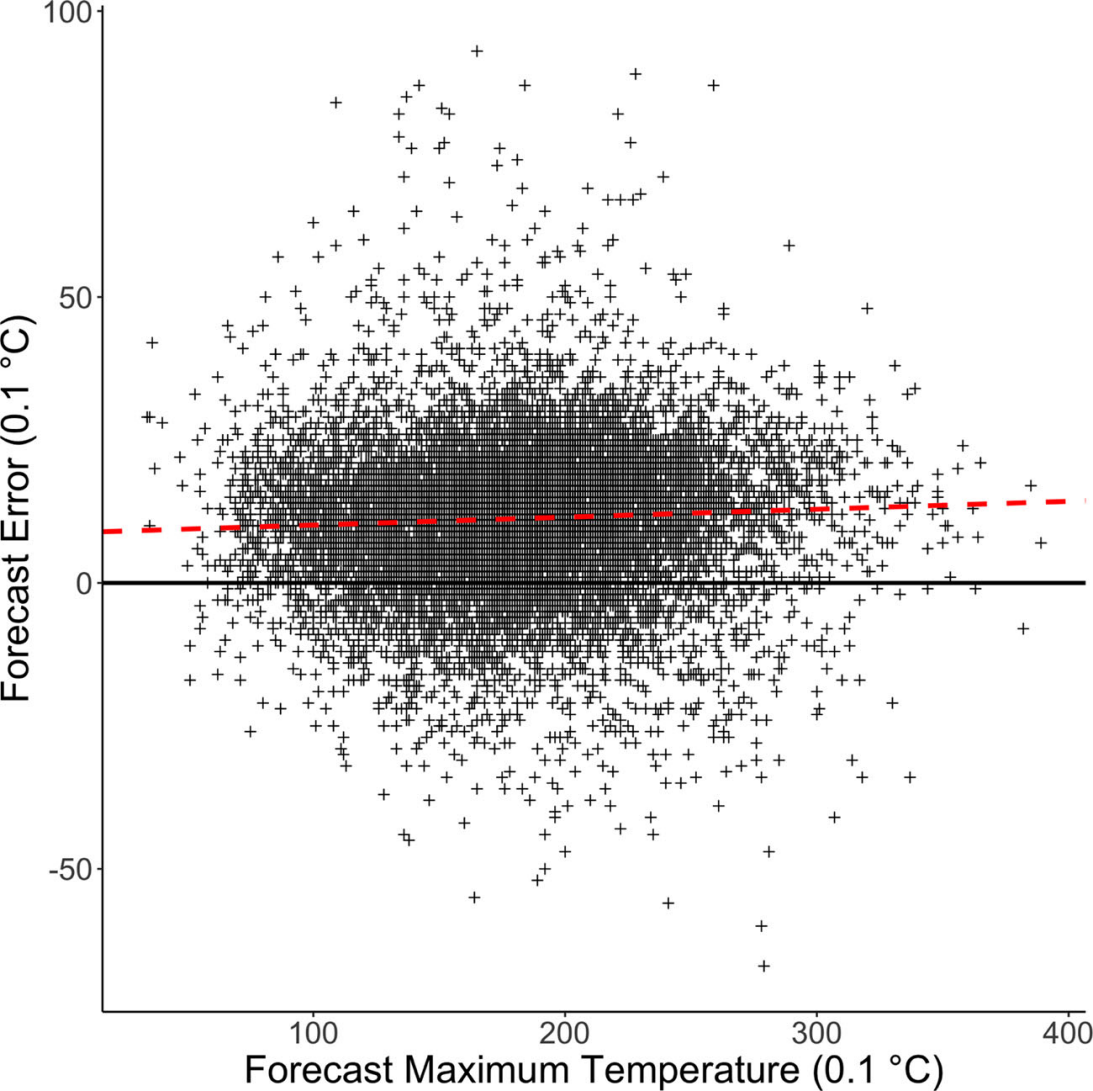
Scatter plot of error of the ECMWF high resolution deterministic run maximum temperature forecast against that deterministic forecast in the warm half year for the years 2011-2019 at De Bilt. The black line indicates a zero error and the red dashed line is the linear regression of the data points.

There are substantial risks related to extremely high temperatures. Consecutive days of high temperatures, i.e. heat waves, lead to higher mortality, especially among older people. Besides high temperatures can cause train rails to expand and thereby potentially disrupt the train system. Additionally, in the absence of rain they likely cause severe droughts as seen in 2018 in The Netherlands, which has had large consequences for nature areas and agriculture. The Royal Netherlands Meteorological Institute (KNMI) issues alarms for persistent warm weather. To design a good alarm system it is essential to have good quality weather forecasts. One of the most used ensemble models, the European Centre for Medium-Range Weather Forecasts (ECMWF) ensemble model, has a negative bias in the maximum temperature forecast. As an illustration, Figure 3 shows the forecast bias for data observed at weather station de Bilt where KNMI is located. For accurate forecasts, this bias needs to be corrected for. This can be easily done by estimating the linear relation between the forecasts and the observations. Although this quickly improves the maximum temperature forecast, this leaves unused a vast amount of forecast data for other weather types. We will show that using a wide range of potential covariates, the maximum temperature forecasts are improved further than by a simple bias correction. By performing the variable selection we then also investigate in more detail what effect different covariates have on the forecast distribution estimated using the random forest model.

We use maximum temperature observed at seven stations spread across The Netherlands, namely Den Helder, Schiphol, De Bilt, Eelde, Twente, Vlissingen and Maastricht (http://projects.knmi.nl/klimatologie/daggegevens/selectie.cgi). The focus is on high temperatures, hence we consider only observations from mid-April until mid-October. In total, we look at 9 years of data ranging from 2011 to 2019.

As covariates we use the output of the ECMWF model, which contains a 51 member ensemble and a higher resolution deterministic run. These forecasts are initiated two times a day, at 00 UTC and at 12 UTC, but here we use only forecasts of the latter run. We define the lead time of the forecast as the time difference between the start of the day for which the forecast is valid and the initiation time of the forecast. For this analysis we will consider forecasts with lead times equal to 36 + 24*k* hours for *k* = 0, 1, 2, 3, 4, 5. The ensemble contains 51 exchangeable members and in order to use them we compute a set of summary statistics from the ensemble. These summary statistics are the mean, standard deviation, quantiles and number of ensemble members exceeding a pre-specified threshold. For the quantiles in our application we choose the 25%, 50% and 75 % quantiles. Thresholds are chosen as to extract different types of information from the ensemble relative to the weather phenomenon itself. For cloud cover we use three thresholds, 20 %, 50 % and 80 % of cloud cover to create variables measuring probabilities of a few to no clouds, partly clouded weather and clouded weather.

Apart from the forecasts for maximum temperature and cloud cover, we consider other covariates including forecasts for daily average temperature at 2 m, dew point temperature, minimum temperature, daily average wind speed and daily accumulated precipitation. For long lead times, predictability of these typical weather phenomena decreases, but the range of predictability of for example flow pattern at 500 hPa extends much further. Therefore the first three principal components flow pattern at 500 hPa over Europe are also used as predictors [12]. Note that these covariates are the same for each station.

For the response variable we consider the forecast error, which we obtain by subtracting the deterministic forecast run from the observed maximum temperature. By doing so, the seasonality in the temperature is largely reduced. In Figure 3, the forecast error is clearly visible as the distance between the red linear regression line and the *x*-axis is rather large. Additionally it is clear that the spread of error changes as a function of the deterministic forecast. A possible explanation is that there still remain seasonality effects that are not taken care of by a simple linear effect. Therefore, also the sine and cosine of the day of the year with a period of one year and half a year are included as two predictors. In total this gives us 71 covariates. For a given lead time an observation on a given day is denoted by (Y,X), with *Y* the error of the deterministic run and X the 71 dimensional covariate vector.

In what follows, we will explore 3 methods, quantile random forests as in [1] with all variables, quantile random forests with variables selected by our forward variable selection and Non-homogeneous Gaussian Regression (NGR) [8]. This third method is known in the meteorology literature as an EMOS (Ensemble Model Output Statistics) method and is used as a standard approach in post-processing. The NGR method assumes the data follow a Gaussian model,

$$Y|X=x∼N\left(x^{T}\beta ,\exp(x^{T}\gamma )\right).$$

The parameters *β* and *γ* are then estimated by maximum likelihood. For this model, we select variables based on the Bayesian Information Criterion (BIC) by a forward and backward stepwise approach.

**Figure 4.**
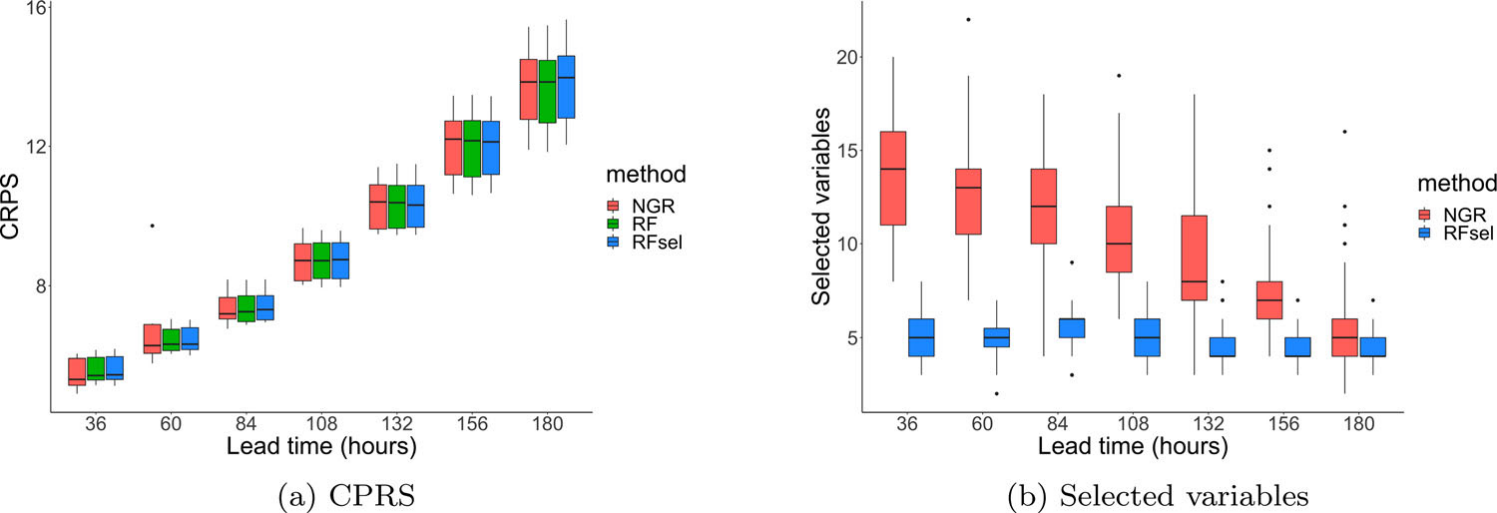
(Left panel) the CRPS risk against leadtime where the boxplots contain the CRPS risk for each station. (Right panel) the number of selected variables against leadtime.

For each station and lead time, we fit a separate model. The models are estimated with a 9-fold cross validation, each time leaving out a single year. In Figure 4(a) the CRPS risk is shown as a function of lead time, where the box-plots contain the CRPS risk for all stations. Then in Figure 4(b) the number of selected variables is shown for our method and NGR, where we leave out the random forest with all 71 variables.

**Figure 5.**
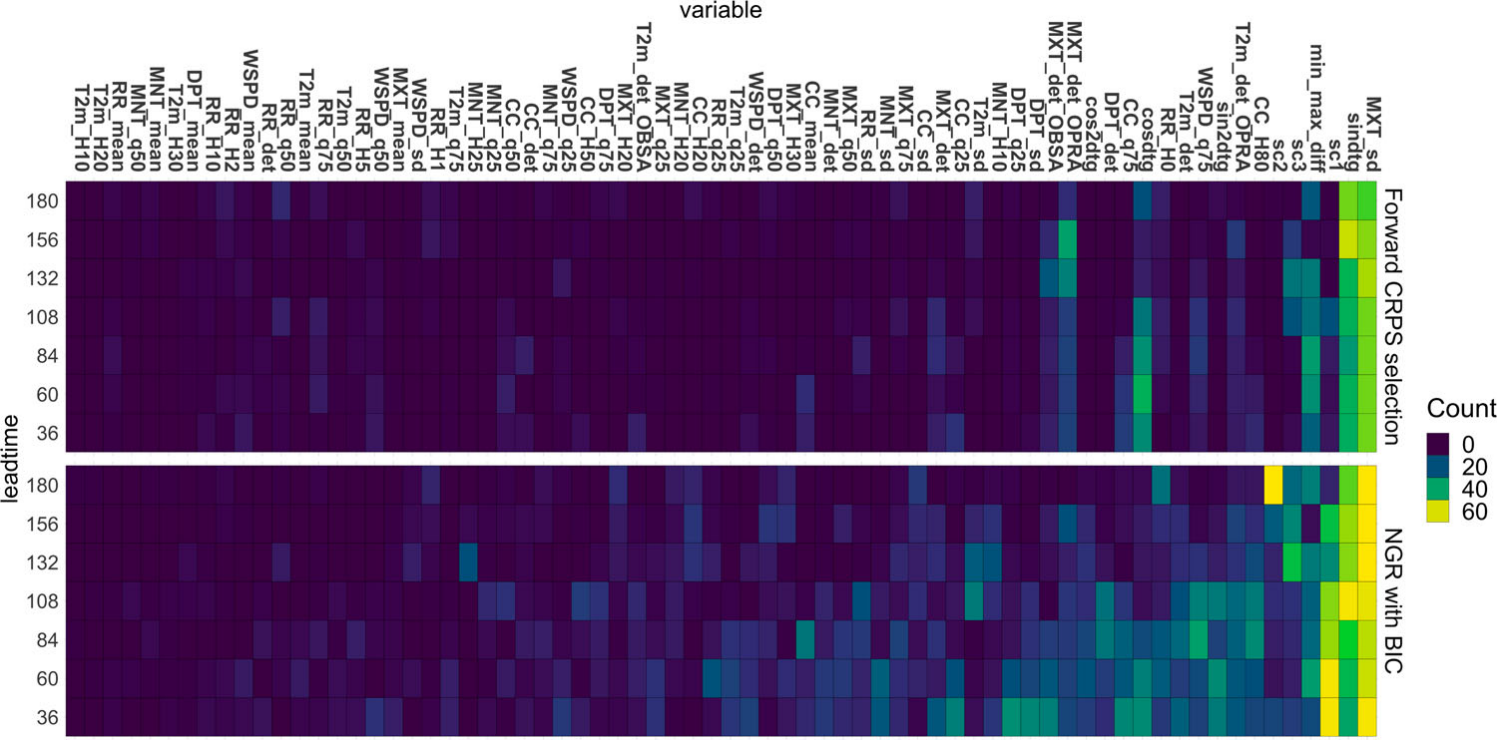
Selected variables for each lead time. All cross validations and all stations have been aggregated where the maximum number of times a variable can be selected is 63.

Based on the CRPS, all methods perform comparably. This is also confirmed by other verification measures such as reliability diagrams, quantile reliability diagrams and probability integral transform histograms, which are not shown in this paper. A selection of these diagrams is shown in the appendix. The interesting part comes from the number of selected variables. Our method selects a small portion (less than 10%) of covariates, substantially less than NGR. We investigate this further by considering which variables are selected. The result is visualized in Figure 5. For each lead time, the color indicates the frequency of a covariate being selected by 63 estimated models (7 locations and 9 cross-validation sets per location). An extremely important variable would be selected all 63 times.

Yellow boxes correspond to a few variables that are always selected. But the number of light blue boxes is much smaller for our method compared to NGR. From this we conclude that our method selects fewer variables and it also selects similar variables for different stations. This suggests that our variable selection method is more robust compared to the NGR method for short lead times, where a diverse set of variables is selected.

**Figure 6.**
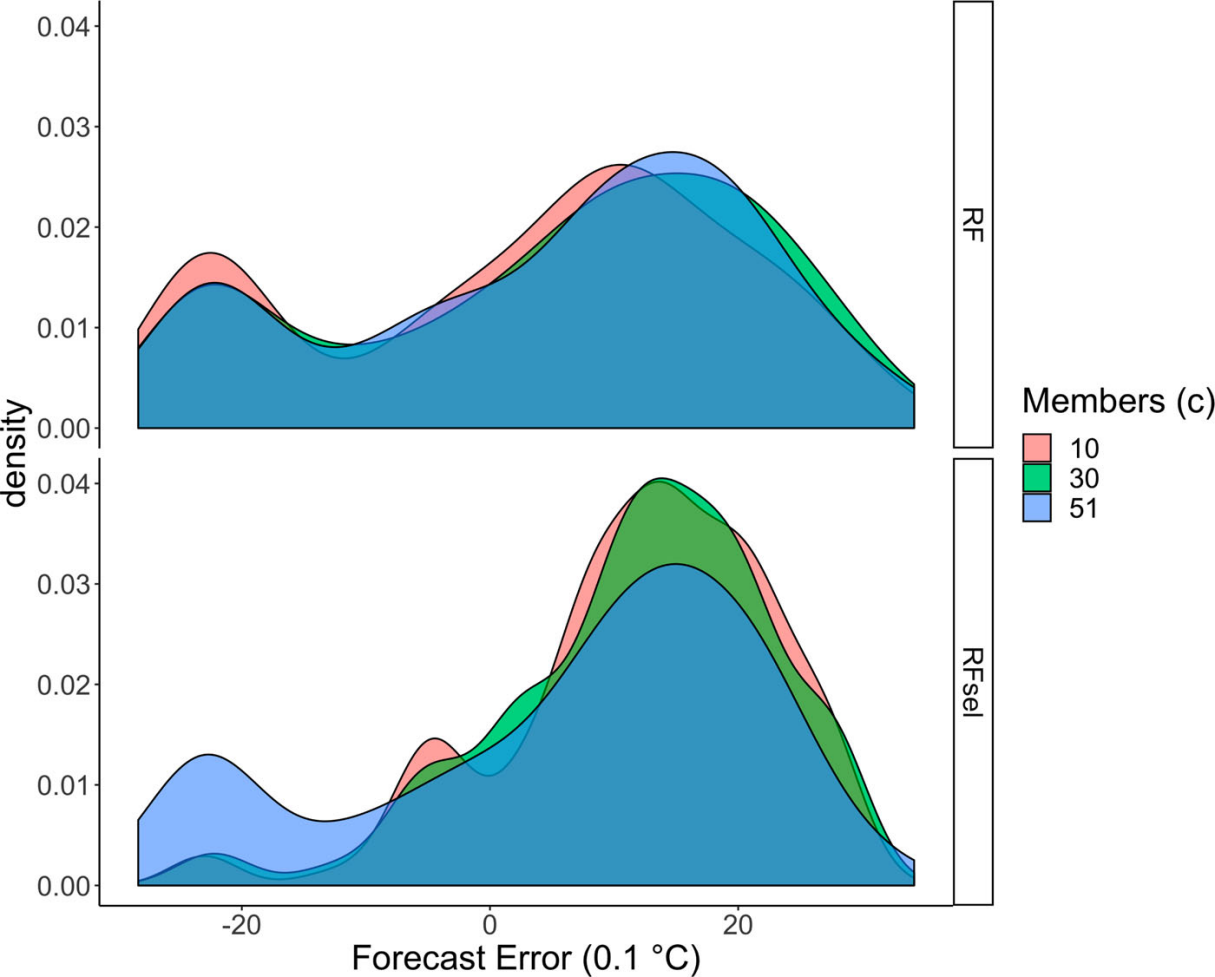
The conditonal distribution of the forecast error, based on estimated models for De Bilt with lead time 36 h. Different colors indicate different values of cloud cover while the values of other covariates are fixed to be the same as that for 31-05-2018 at de Bilt. Top figure for the random forest model with all variables and the bottom figure for the random forest model with the selection of variables.

The main variables that our method selects are the sine of the day of the year, the standard deviation of the ensemble forecast and variables related to cloud cover. Since our procedure typically selects a small set of variables, it is then feasible to interpret the estimated model. For instance, to investigate how a selected covariate, say $X_{j}$ influences the forecast distribution of *Y*, one can compare the conditional distribution of *Y* given different values of $X_{j}$ while the other covariates denoted by $X_{\left(-j\right)}$ are kept the same. We consider *Y*, the forecast error at de Bilt with lead time 36 h and $X_{j}$ the cloud cover, which is the number of ensemble members with cloud cover exceeding 50%. The values of other covariates are fixed the same as the data of 31-05-2018 at De Bilt, denoted by $X_{\left(-j\right)}=x_{\left(-j\right)}^{*}$. Figure 6 shows the conditional density of *Y* given $\left(X_{j}=c,X_{\left(-j\right)}=x_{\left(-j\right)}^{*}\right)$, where different colors indicate three different values of *c*. Note that all 51 ensemble members exceed 50% cloud cover. As shown in the lower panel of Figure 6, cloud cover clearly has an effect based on the estimation of our method: *c* = 51 yields a bimodal distribution while *c* = 10 leads to a unimodal distribution. This suggests that in this configuration, higher cloud cover implies a higher chance for a negative forecast error (left mode in the plot). However, the distributions obtained by random forest (without variable selection) are very similar; see the upper panel of the figure. This is because that there are other covariates correlated to cloud cover, and these covariates still indicate that there is a high cloud cover even when the number of ensemble members exceeding 50% could cover is set to 10. In other words, changing the value of a single variable in a random forest with many correlated covariates is *not* interpretable. Such a random forest model fails to capture the effect of a signal variable.

## 6. Summary and discussion

In this paper, we have proposed a general framework for a forward variable selection with respect to a loss function. We show in population sense that under an independence assumption between covariates and by choosing the continuous ranked probability score as loss function that the forward selected variables form the correct set with respect to the CRPS risk functional. Applying the method in a random forest set-up, we show that the out-of-bag samples can be efficiently used to asses predictive performance. The main difficulty in the procedure is determining the stopping time, that is when selecting more variables does not add in predictive performance. Due to randomness and the inherent greedy variable selection procedure in the random forest algorithm this can not be determined by the calculated predictive performance. Instead in a single forward selection step we use the predictive performance of each possible set to construct a test to detect increasing predictive performance. The procedure then stops a null hypothesis of non increasing predictive performance can not be rejected. We show that this test is consistent.

With a simple simulation study we show that our variable selection method, compared to a backward selection based on a permutation importance measure, is more capable of discriminating between signal variables and noise variables. This improvement is shown for various sample sizes and correlations between the covariates. Additionally we show that similar results are obtained in a high dimensional setting where *d*>*n*.

In an application on post-processing maximum temperature, our method shows consistency in the number of selected variables and in the variables being selected over several stations. Moreover, our method selects less than 10 % of the covariates and still attains the same predictive power as the quantile random forest with all covariates. Further, it is easier to interpret our resulting model, due to the largely reduced number of covariates. Without variable selection, it is hardly possible to analyze the effect of a single covariate in a random forest model when it is heavily correlated to other covariates. In our data example, in the presence of thick cloud cover, our random forest model indicates that there is a higher risk of over forecasting (lower panel of Figure 6) instead of under-forecasting which was indicated by Figure 3.

There are two interesting directions for future research. First, the theoretical results in Sections 2 and 3 are derived under the assumption that the covariates are independent. However, the ability of our method to select signal variables from a correlated setting is evidenced by our simulation study and data application. It is interesting to investigate such a setting. Second, we focus in this paper on how this forward method behaves for the CRPS, but the mathematical set-up in Section 2 is much more general and allows to select variables with respect to other loss functions. It would be interesting to extend the current results to a more general set of loss functions.

## Supplementary Material

Supplemental Material
