# Supplementary material for: Forward variable selection for random forest models
Source: J Appl Stat. 2022 Jul 18;50(13):2836–56. doi: 10.1080/02664763.2022.2095362 (PMC10503461; doi:10.1080/02664763.2022.2095362)
Supplement: Supplemental Material [file CJAS_A_2095362_SM0671.pdf]

Supplemental Material to "Forward variable selection for random forest models"

Jasper Velthoen[a], Juan-Juan Cai[b] and Geurt Jongbloed[a]

[a]Department of Applied Mathematics, Delft University of Technology, Mekelweg 4, 2628CD Delft, The Netherlands
[b]Department of Econometrics and Data Science, Vrije Universiteit Amsterdam, De Boelelaan 1105, 1081 HV Amsterdam, The Netherlands

ARTICLE HISTORY
Compiled May 1, 2022

Appendix A. CRPS calculations

Here we show for an observation y and a distribution function F that the CRPS calculated from the quantile perspective as well as from the distribution function perspective are equivalent as shown in [1], i.e we show that

$$2\int_0^1 \rho_\tau(y - F^{-1}(\tau))\mathrm{d}\tau = \int_{-\infty}^{\infty} (I(y \leq z) - F(z))^2 \mathrm{d}z. \tag{A1}$$

We have,

$$\begin{aligned}
2\int_0^1 \rho_\tau(y - F^{-1}(\tau))\mathrm{d}\tau &= 2\int_0^1 (I(y \leq F^{-1}(\tau)) - \tau)(F^{-1}(\tau) - y)\mathrm{d}\tau \\
&= 2\int_{-\infty}^{\infty} (I(y \leq z) - F(z))(z - y)f(z)\mathrm{d}z \\
&= -(I(y \leq z) - F(z))^2(z - y)\big|_{-\infty}^{\infty} + \\
&\int_{-\infty}^{\infty} (I(y \leq z) - F(z))^2 \mathrm{d}z \\
&= \int_{-\infty}^{\infty} (I(y \leq z) - F(z))^2 \mathrm{d}z
\end{aligned}$$

Here we use a substitution in the second line of $\tau = F(z)$ and in the third line we apply integration by parts.

CONTACT Jasper Velthoen. Email: j.j.velthoen@tudelft.nl

Appendix B. Justification on the stopping criterion in Section 3.4

In this section, we provide some mathematical justification for the stopping criterion introduced in Section 3.4. We formulate the problem under the framework of a hypothesis test. Let $I_0 \subset \{1, \ldots, d\}$ and $I_1 = I_0 \cup \{j_0\}$, where $j_0 \notin I_0$. One can think I_0 as $\hat{J}_{j-1}$ and I_1 as $\hat{J}_j$ at the j-th step of selection. We wish to test

$$\begin{cases} H_0: & R(I_0) - R(I_1) = 0 \\ H_A: & R(I_0) - R(I_1) > 0. \end{cases}$$

We propose the following test statistics:

$$W = \sum_{q \notin I_1} \mathbb{I}(\hat{R}(I_0 \cup \{q\}) - \hat{R}(I_1 \cup \{q\}) > 0). \tag{B1}$$

Under the null hypothesis, W approximately follows the $\text{Bin}(M, 0.5)$ distribution, where $M = d - |I_1|$. Our stopping criterion is equivalent to reject H_0 if $W > C_{1-\alpha}$, where $C_{1-\alpha}$ is the $1 - \alpha$ quantile of $\text{Bin}(M, 0.5)$ and α is the significance level of the test. The consistency of this test is established in the theorem below.

Note that the theorem is based on the assumptions that the covariates are independent as in Theorem 2.1 and the conditional quantile estimators by random forest are consistent. The consistency is established for fixed d, thus not including the case $d = d(n) \to \infty$. This result is just a proof of concept to support the selection procedure.

Theorem B.1. *Assume that for any $\tau \in (0, 1)$, as $n \to \infty$,*

$$\frac{1}{n} \sum_{i=1}^{n} \left| \hat{Q}^{\mathcal{F}_i}(\tau | \mathbf{X}_i^J) - Q(\tau | \mathbf{X}_i^J) \right| \xrightarrow{p} 0, \tag{B2}$$

where $J = I_0 \cup \{q\}$ or $J = I_1 \cup \{q\}$, $q \notin I_1$. Then, under the assumptions of Theorem 2.1 and that $M > C_{1-\alpha}$,

$$\mathbb{P}(W > C_{1-\alpha}) \to 1 \quad \textit{Under hypothesis } H_A, \tag{B3}$$

as $n \to \infty$.

It suffices to prove that under H_A, as $n \to \infty$

$$\mathbb{E}[W] \to \omega_0,$$

where $\omega_0 > C_{1-\alpha}$.

Denote $I_q := I_0 \cup \{q\}$ and $K_q := I_1 \cup \{q\}$, $q \notin I_1$. Then, we have

$$
\begin{aligned}
&\hat{R}(I_q) - \hat{R}(K_q)\\
=&\frac{2}{n}\sum_{i=1}^{n}\left(\int_0^1 \rho_\tau(Y_i - \hat{Q}^{\mathcal{F}_i}(\tau|\mathbf{X}_i^{I_q}))\mathrm{d}\tau - \int_0^1 \rho_\tau(Y_i - \hat{Q}^{\mathcal{F}_i}(\tau|\mathbf{X}_i^{K_q}))\mathrm{d}\tau\right)\\
=&\frac{2}{n}\sum_{i=1}^{n}\left(\int_0^1 \rho_\tau(Y_i - \hat{Q}^{\mathcal{F}_i}(\tau|\mathbf{X}_i^{I_q}))\mathrm{d}\tau - \int_0^1 \rho_\tau(Y_i - Q(\tau|\mathbf{X}_i^{I_q}))\mathrm{d}\tau\right)\\
&+\frac{2}{n}\sum_{i=1}^{n}\left(\int_0^1 \rho_\tau(Y_i - Q(\tau|\mathbf{X}_i^{K_q}))\mathrm{d}\tau - \int_0^1 \rho_\tau(Y_i - \hat{Q}^{\mathcal{F}_i}(\tau|\mathbf{X}_i^{K_q}))\mathrm{d}\tau\right)\\
&+\frac{2}{n}\sum_{i=1}^{n}\left(\int_0^1 \rho_\tau(Y_i - Q(\tau|\mathbf{X}_i^{I_q}))\mathrm{d}\tau - \int_0^1 \rho_\tau(Y_i - Q(\tau|\mathbf{X}_i^{K_q}))\mathrm{d}\tau\right)\\
=:&S_1 + S_2 + S_3.
\end{aligned}
$$

Applying the Knight's identity, $\rho_\tau(u-v) - \rho_\tau(u) = -v(\tau - I(u<0)) + \int_0^v (I(u \leq s) - I(u \leq 0))\mathrm{d}s$, which implies that $|\rho_\tau(u-v) - \rho_\tau(u)| \leq 2|v|$, we have

$$
|S_1| \leq \frac{4}{n}\sum_{i=1}^{n}\int_0^1 \left|\hat{Q}^{\mathcal{F}_i}(\tau|\mathbf{X}_i^{I_q}) - Q^{\mathcal{F}_i}(\tau|\mathbf{X}_i^{I_q})\right| \mathrm{d}\tau \xrightarrow{p} 0,
$$

by (B2). The same result holds for S_2.
Observe that S_3 is the sample mean of I.I.D. random variables with expectation $R(I_q) - R(K_q)$. Applying law of large number, $S_3 \xrightarrow{p} R(I_q) - R(K_q)$. Combing with the results for S_1 and S_2, we have

$$
\hat{R}(I_q) - \hat{R}(K_q) \xrightarrow{p} R(I_q) - R(K_q).
$$

Under H_a, $j_0 \in J^*$, thus, by the proof for Theorem 2.1, for all $q \notin I_1$,

$$
R(I_q) - R(K_q) > 0.^1
$$

This implies that

$$
\mathbb{E}[W] = \sum_{q \notin \hat{J}_j} \mathbb{P}\left(\hat{R}(I_q) - \hat{R}(K_q) > 0\right) \to M > C_{1-\alpha}.
$$

Appendix C. Calibration of forecasts for lead time 60 and station De Bilt

Figure C1 shows a histogram of the $\hat{F}(Y)$ where $\hat{F}$ is the forecast distribution for observation Y. If F is calibrated the histogram should look like the histogram based on standard uniform random variable.

Figure C2 shows reliability diagrams. Let t be a threshold and define $p = \hat{F}(t)$ and $I = I(Y \leq t)$ for each forecast. A reliability diagram bins the probabilities p in

[1] Obviously under H_0, $R(I_q) - R(K_q) = 0$.

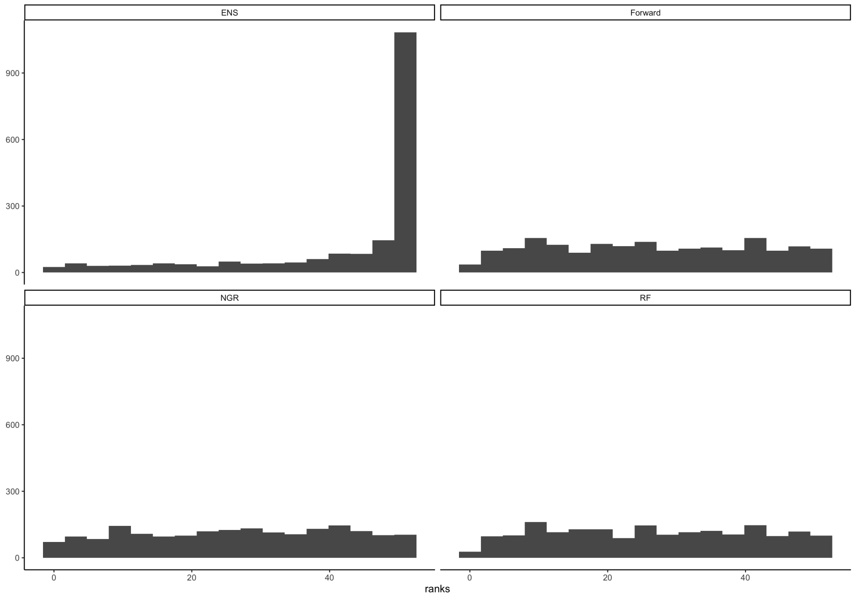

Figure C1.: Rank histograms for lead time 60 h and station De Bilt. For forward selection, NGR, random forest and the raw ensemble forecast

equally sized bins. The average indicator I should be the same as the average p. Hence plotting these averages they should be approximately on the identity line; for detailed explanation we refer to [2].

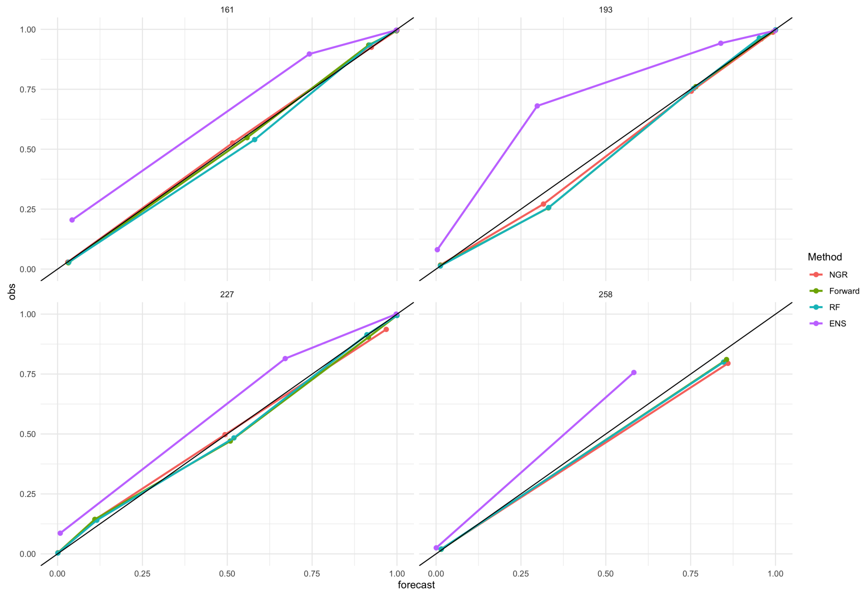

Figure C2.: Reliability diagrams with thresholds equal to the $0.25, 0.5, 0.75$ and 0.9 observational quantiles for lead time 60 and station De Bilt. Methods compared are: forward selection, NGR, random forest and the raw ensemble forecast

Figure C3 shows quantile reliability diagrams. Let τ be a probability level and $\hat{Q}$ the forecast quantile function. Define $q = \hat{Q}(\tau)$ for each forecast. A quantile reliability

diagram bins the quantiles q in equally sized bins. The τ quantile of observation Y should be the same as the average q. Hence plotting these against each other should be approximately on the identity line; for detailed explanation we refer to [3].

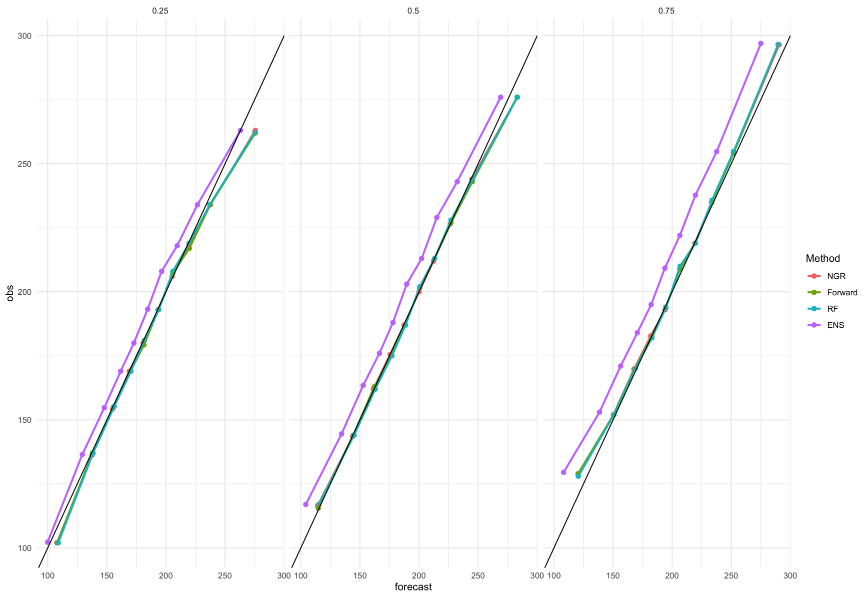

Figure C3.: Quantile reliability diagrams for quantile levels equal to $0.25, 0.5$ and 0.75 for lead time 60 and station De Bilt. Methods compared are: forward selection, NGR, random forest and the raw ensemble forecast

References

[1] F. Laio, S. Tamea, Verification tools for probabilistic forecasts of continuous hydrological variables, Hydrology and Earth System Sciences 11 (4) (2007) 1267–1277.

[2] D. S. Wilks, Statistical methods in the atmospheric sciences, Vol. 100, Academic press, 2011.

[3] S. Bentzien, P. Friederichs, Decomposition and graphical portrayal of the quantile score, Quarterly Journal of the Royal Meteorological Society 140 (683) (2014) 1924–1934.
